# Diet influences proliferation and stability of gut bacterial populations in herbivorous lepidopteran larvae

**DOI:** 10.1371/journal.pone.0229848

**Published:** 2020-03-13

**Authors:** Charles J. Mason, Abbi St. Clair, Michelle Peiffer, Elena Gomez, Asher G. Jones, Gary W. Felton, Kelli Hoover

**Affiliations:** Department of Entomology, The Pennsylvania State University, University Park, PA, United States of America; Zhejiang University, CHINA

## Abstract

Animals have ubiquitous associations with microorganisms, but microbial community composition and population dynamics can vary depending upon many environmental factors, including diet. The bacterial communities present in caterpillar (Lepidoptera) guts are highly variable, even among individuals of a species. Across lepidopteran species, it is unclear if the variation in their gut bacterial communities is due to ingested bacteria with diets or responses of gut bacteria to their diet. In this study, we aimed to understand whether bacteria establish and persist in the lepidopteran gut or just pass through the gut with food. We also examined whether bacterial establishment in lepidopteran guts depended on diet. We conducted a series of experiments using axenic and gnotobiotic insect rearing methods to address these objectives. We found that bacteria were established and maintained without replacement through the larval instars of the fall armyworm (*Spodoptera frugiperda*) and corn earworm (*Helicoverpa zea*). Gut bacterial titers increased when larvae were fed gamma-irradiated corn leaves but decreased when fed a wheat germ artificial diet. However, bacterial titers of larvae fed on a pinto bean artificial diet were similar to those consuming intact plants. We also observed that microbial titers of fall armyworm and other folivorous larvae were positively related to the host body size throughout larval development. Collectively, these results suggest that the populations of bacteria present in caterpillar guts are not simply a transient community passing through the system, but rather are a dynamic component of the caterpillar gut. Sensitivity of bacterial populations to the type of diet fed to lepidopterans suggests that not all diets are equally useful for reducing variance in community structure and interpreting insect-microbe interactions.

## Introduction

Animals have ubiquitous associations with microorganisms, with these relationships spanning differing degrees of association and sensitivities to environmental changes. Diets are among the factors that can alter host–microbe interactions and are capable of having both short- and long-term impacts on animal gut microbial community assemblages, affecting both taxonomic and functional associations [1–5]. Within invertebrates, lepidopteran larvae are particularly sensitive to diet- and environment-driven changes to gut bacterial assemblages. Several studies have described the communities associated with various members of this insect order [6–17], with a major connection being that environment and diet can influence bacterial community composition [18]. The bacterial communities present in lepidopteran guts also tend to be variable, dynamic, and functionally redundant [18–20]. These characteristics create some inherent challenges in evaluating insect–microbe interactions in lepidopteran systems, especially compared to insects that harbor more stable microbial communities [21,22].

Despite the growing body of information regarding microbial interactions in Lepidoptera, there remains a key question that has not yet been answered: how transient are their gut microbial populations? Currently, it is unclear if repeated bacterial recruitment is needed to maintain populations in lepidopteran guts like in some other systems [23] and if the bacteria found in the digestive tract is simply a reflection of microbes associated with their food [17,18]. Given the alkaline environment of lepidopteran guts (pH 8–11) [24,25] and the rapid transit of food though the gut, determining whether external bacteria establish and proliferate, or are transients passing through the digestive system, can provide insights into factors that influence bacterial community assembly and host–microbe interactions. Although artificial diets can be useful for investigating insect–microbe interactions, such diets are not representative of the plant foliage that caterpillars consume in nature. Not only do artificial diets provide unrealistic quantities of soluble carbohydrates, proteins, and available fats, but they lack the physical properties, elemental complexity, and secondary metabolite components that are commonly associated with plant foliage. Understanding if bacterial communities establish, whether they decline, persist, or proliferate, and if these processes differ between diets are important in delineating the impacts and directionality of these relationships.

In this study, we addressed how diets impact the establishment and proliferation of bacterial populations in folivorous lepidopteran guts. We conducted a series of manipulative experiments using axenic rearing and gnotobiotic techniques of larvae to produce defined bacterial gut communities containing ecologically relevant bacterial isolates. Two of the larval diets were artificial and semi-artificial and made in the laboratory, while the third was from greenhouse-grown plants. To eliminate the introduction of plant-associated microbes to the herbivore gut, we employed gamma irradiation to fully sterilize the plant foliage we fed to the larvae. This enabled us to produce a manipulative system to test the effects of diet on the population dynamics within an insect gut, while eliminating the major confounding factor that occurs with repeated introductions of bacteria associated with food.

Fall armyworm (*Spodoptera frugiperda*) and corn earworm (*Helicoverpa zea*) are two of the most economically important lepidopteran species in North America. Both species are highly polyphagous, have a partially overlapping host range, and are amenable to laboratory rearing on artificial diets. Like most lepidopterans [18,22], the microbial communities of these species are simple and dynamic. In both species, field-collected larvae tend to be populated by members in the Enterobacteriaceae, whereas laboratory-reared larvae have gut communities primarily composed of *Enterococcus* [15,26]. Comparable microbial community membership and compositions have been observed for other *Spodoptera* and *Helicoverpa* species, with various Firmicute and Gammaproteobacteria isolates comprising the most abundant taxa [9,11,12]. Diet has been suggested as a key driver of gut bacterial composition for both *Spodoptera* and *Helicoverpa*, and similar patterns have been observed and suggested for additional species [18].

## Methods

### Insect rearing and diet conditions

Fall armyworm and corn earworm colonies were obtained from Benzon Research Laboratories (Carlisle, PA, USA) and maintained in the laboratory. Eggs were collected from mating containers of both species and surface sterilized with 2.5% bleach before use in experiments. Larvae were hatched from surface sterilized eggs in autoclaved containers and feedings were completed in a laminar flow hood under aseptic conditions. Inoculations with bacteria and rearing of individuals were completed in 22.5 mL plastic cups that were sterilized with 70% ethanol under UV light. Larvae were maintained in a 28°C growth chamber under a 16:8 light: dark cycle.

Artificial diets were formulated as previously described [27–29] with some modifications. The wheat germ diet contained (g L^-1^): casein (33), dextrose (33), wheat germ (28), Wesson salt mix (9), ascorbic acid (5), cellulose (4.7), sodium alginate (4.7), cholesterol (1.8), methyl paraben (1.5), sorbic acid (0.5), choline chloride (1.1), agar (26), and wheat germ oil (5 mL L^-1^). Pinto bean diet contained (g L^-1^): dried pinto beans (100), fortified yeast (21.3), ascorbic acid (4), methyl paraben (1.5), sorbic acid (0.67), propionic acid (0.5), agar (9), and lepidopteran vitamin mix (3.3) (Bioquip). Artificial diets commonly contain antimicrobials to prevent food spoilage and can range from being highly antibacterial (e.g. streptomycin) to relatively benign to particular microbes. Methyl paraben, sorbic acid, and propionic acid can be antimicrobial, especially against fungi, and are common in artificial diets. Artificial diets were autoclaved after mixing and poured into sterile petri dishes.

Corn (*Zea mays* B73 genotype) plants were grown in the greenhouse in a 2:1 mixture of topsoil and potting media. The greenhouse was maintained at ~28°C and 16:8 light: dark cycle. Corn was maintained until the V5-6 stage and then cut into squares. Foliage was sterilized with gamma irradiation at previously described [30] to ensure no microbes were transferred to feeding larvae. Gamma irradiation was performed at The Pennsylvania State University Radiation Science and Engineering Center Gamma Irradiation Facility. Corn leaves were subjected to a 15 kilogray dose from radioactive Cobalt-60, which has two high energy gamma rays (1.17MeV and 1.33 MeV).

We used a combination of techniques to address the presence of viable bacteria in our system. We used RT-PCR of partial 16S-rRNA to determine if the irradiation and sterilization steps were suitable for prevention of bacterial establishment. We then verified these results with plate culturing and enumeration to determine cell viability and population dynamics of gut bacteria within our treatments.

### Verification of axenic state of insects

Due to the issues surrounding plastid (e.g. chloroplast) interference with 16S-rRNA amplification of genomic DNA [31], we used culturing and RNA analyses to evaluate the viability of microbes associated with plant diets and insects, respectively. Corn leaves that were gamma-irradiated, collected from the greenhouse, and collected from the field were homogenized and plated onto nutrient-rich 2×YT medium [32] to determine differences in viable microbial titers by counting colony forming units (CFUs).

We compared the axenic status of fall armyworm larvae with those treated with a mixed bacterial community. Sterilized fall armyworm neonates were maintained in diet cups on sterile wheat germ diet until the second instar. Insects were fed bacterial isolates in phosphate buffered saline (pH 7; PBS) applied to a diet cube as previously described [30]. To generate insects containing bacteria, larvae were fed a mixed bacterial community that was prepared from frass (feces) collected from insects feeding on corn leaves from the field. Frass was homogenized in PBS and particulates were separated from the bacterial fraction using differential centrifugation. Insects were reared on gamma-irradiated corn leaves until the beginning of the 6^th^ instar. Midguts from axenic and mixed community insects were dissected and a portion plated onto 2×YT medium with the remainder being flash frozen for RNA extraction. No CFUs were detected for axenic larvae.

RNA was extracted from axenic and mixed community fall armyworm guts using Trizol (Thermo-Fisher Scientific) followed by a lithium chloride precipitation to remove contaminating DNA. Complementary DNA (cDNA) was synthesized from RNA using the High Capacity cDNA reverse transcription kit (Applied Biosystems). PCR targeting the bacterial 16S was conducted using GoTaq Green Master Mix (Promega) with the 16S primers 515F (3’-GTGCCAGCMGCCGCGGTAA-5’) and 806R (3’-GGACTACHVGGGTWTCTAAT-5’). PCR was performed on the fall armyworm housekeeper gene elongation factor 1a (F: 3’- CGATTTCACAGCACAGGTCATC-5’; R: 3’- CAGGCAATGTGGGCTGTGT-5’) using the following conditions: 94°C 2 min; 25 cycles of 94°C 30s, 50°C 30s, 72°C 30s; 72°C 5 min.

### Larval development and bacterial proliferation

Sterilized fall armyworm neonates were maintained in diet cups on sterile wheat germ diet until the second instar. Insects were then fed one of three bacterial isolates in phosphate buffered saline (pH 7; PBS) applied to a diet cube as previously described [30]. Uninoculated control larvae were fed a diet cube to which 50 μL of PBS was applied. We used three bacterial isolates: *Enterobacter sp*. FAW4-1, *Klebsiella sp*. FAW8-1, and *Enterococcus sp*. FAW13-5. These isolates were isolated from field-collected fall armyworm; these taxa comprise significant portions of the gut bacterial community as determined using 16S-rRNA amplicon analyses [15]. These isolates also have distinct morphologies when grown on media, which allowed for visual identification during recovery. Diet cubes were inoculated with 50 μL of buffer containing approximately 10^8^ cells of each bacterial isolate. Larvae were fed on the treated diet cube through the second and into the third instar (approximately 2 d). For this and subsequent experiments, bacteria were only provided at this interval, with no additional inoculations occurring on any of the diets.

Upon molting to the third instar, larvae were transferred to individual cups containing sterile wheat germ diet or sterile corn leaves. The wheat germ diet was not replaced during the experiment, while the corn diet was replaced every 48 h. A subset of larvae from each bacterial treatment was harvested at the third, fourth (3 d on diet treatment), and fifth larval (5 d on diet treatment) instar to evaluate bacterial populations for each bacterial treatment. In order to enumerate bacterial populations, larvae were surface sterilized for 30 s in 70% ethanol, rinsed in sterile distilled water, and dried on autoclaved paper towels. Due to the size of the larvae and to ensure the full guts were assayed, third instar larvae were then immediately homogenized in PBS. For fourth and fifth instar larvae, their midguts were dissected prior to homogenization. We made serial dilutions of the tissue homogenates and counted CFUs by plating on 2×YT medium.

### Effects of different artificial diets on bacterial establishment

To determine if different artificial diets have variable impacts on bacterial proliferation in fall armyworm, we compared wheat germ and pinto bean artificial diets with corn foliage. Fall armyworm were reared from sterile eggs as described previously, orally inoculated through the second instar with *Enterobacter sp*. FAW4-1, and then transferred to the respective diets. Wheat germ and pinto bean diets were provided once, while corn leaves were replaced every 48 h for the duration of the experiment (4 d).

### Insect species and bacterial proliferation

To test whether there were differences in bacterial proliferation between insect species, we compared fall armyworm and corn earworm feeding on wheat germ artificial diet and corn foliage. Surface sterilization and insect rearing were conducted as described above. The experiment was performed using *Enterobacter sp*. FAW4-1, and bacteria were provided through the second instar for both insect species. Larvae were maintained on wheat germ or corn diets until the fourth instar (4 d on diet treatment), after which they were weighed and surface sterilized. Larval guts were dissected in aseptic conditions and bacterial CFUs were enumerated by plating serial dilutions on 2×YT medium.

### Comparison of microbial titers and insect body mass

We compared the body size of various folivores with their respective culturalable microbial populations. For these experiments, we did not use any axenic or gnotobiotic methods, but provided insects with plant foliage. In addition to our focal species of larval fall armyworm and corn earworm, we also assayed larval gypsy moth (*Lymantria dispar*) and Colorado potato beetle (*Leptinotarsa decemlineata*). Fall armyworm and corn earworm were fed greenhouse grown corn, Colorado potato beetle was fed greenhouse grown potato (*Solanum tuberosum*), and gypsy moth was fed field-collected black poplar (*Populus nigra*). For fall armyworm, we collected different sized insects over the course of development, surface sterilized them, and performed dissections. For corn earworm, Colorado potato beetle and gypsy moth, we collected fourth instars, surface sterilized them, and dissected their midguts. Microbial CFUs titers were quantified with serial dilution plating on 2×YT medium.

### Statistical analyses

**Fig 1:**
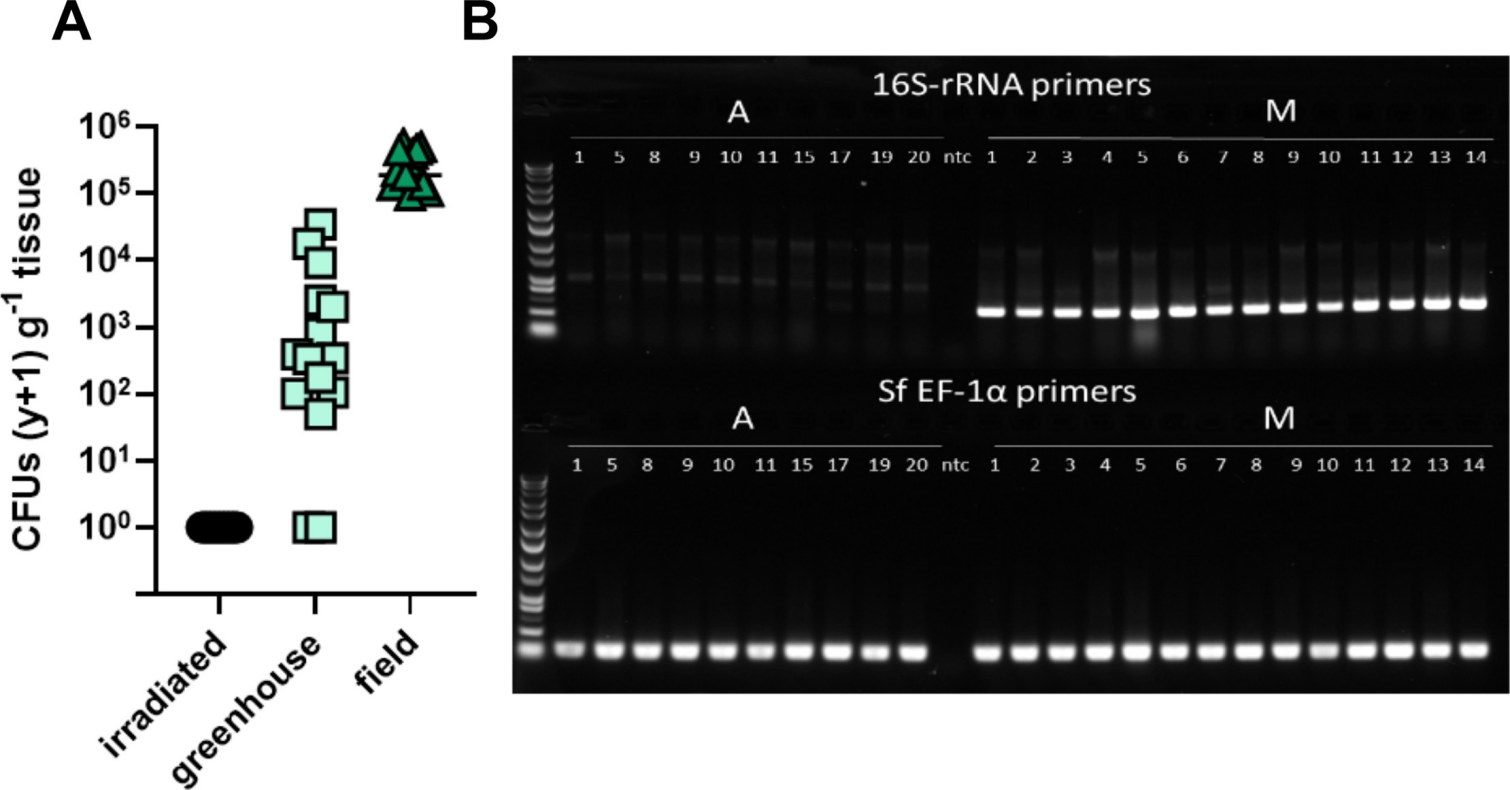
Influence of gamma irradiation of corn leaves on viable microbiota associated with the foliage (A) and expression of 16S-rRNA within the fall armyworm gut (B). Irradiation of greenhouse foliage reduced the number of colony-forming units (CFUs) associated with the foliage (p < 0.001). Fall armyworm larvae that underwent a sterilization procedure (A) did not have detectable microbes in their guts either through plating or through 16S amplification of gut cDNA. In comparison, bacteria were detected in larvae inoculated with bacteria (M) by 16S amplicon sequencing.

Statistical analyses were performed using R-studio unless otherwise noted [33]. CFU counts were log_10_(*y*+1) transformed prior to analyses. Larvae that were sterilized and provided sterile buffer did not possess detectable microbes (see Results Fig 1). The effects of diet (wheat germ diet vs. corn) on isolate establishment (*Enterobacter*, *Klebsiella*, and *Enterococcus*) in fall armyworm were analyzed using non-parametric tests with pairwise comparisons. To evaluate relationships between fall armyworm body size and gut CFUs, linear regression was performed using log_10_ transformed values. Gut bacteria and body sizes for fall armyworm, corn earworm, gypsy moth, and Colorado potato beetle were log_10_ transformed and compared to other previously published data [34]. We compared the resulting CFU (mL^-1^) results with previously published estimates of body size–microbial titers in animals. Kieft and Simmons (2015) reported microbial biomass estimates for various vertebrates and invertebrates. We conducted linear regression using these previously published data and added 95% prediction bands. We then added our data to evaluate if our results for the caterpillar species we tested fell within a similar range as other animal species. Data used the analysis and interpretation of our results are located in the S1 Table.

## Results

### Effects of gamma irradiation on viable corn-leaf microbes and axenic status of insects

There were large differences in microbial titers between field, greenhouse-grown, and greenhouse gamma-irradiated corn leaves (Fig 1A; H = 38.78, p <0.001). Perhaps unsurprisingly, field-grown leaves had the highest titer of culturable microbes (CFUs) compared to the other sources. Greenhouse-grown corn leaves had fewer overall microbes and had greater variation between individuals (Z = 3.43; p = 0.002). The variation we observed was in part due to some leaves having few or no detectable bacteria. As expected, gamma-irradiated foliage had no detectable viable microbiota and provided a consistent sterile food source for our experiments. Axenic larvae and mixed community–inoculated larvae feeding on gamma-irradiated corn leaves followed expected trends. Fall armyworm larvae that underwent a sterilization procedure (axenic; ‘A’in panel) did not have detectable microbes in their guts either through plating or 16S amplification of gut cDNA verified by RT-PCR (Fig 1B). Axenic larvae lacked a band of the expected size (~290bp), while those having gut bacteria (‘M’ in panel) had a band at the expected size (Fig 1B). All larval samples showed amplification of the fall armyworm housekeeper gene elongation factor-1α. These results indicate that through the initial manipulation of our experiments, there were no unculturable bacteria in the gut that we did not detect in culture-based assessments. These results also indicate that gamma irradiation is suitable for reliably eliminating viable microbial titers associated with corn leaves.

### Effect of wheat-germ and corn diets on proliferation of gut microbes

**Fig 2:**
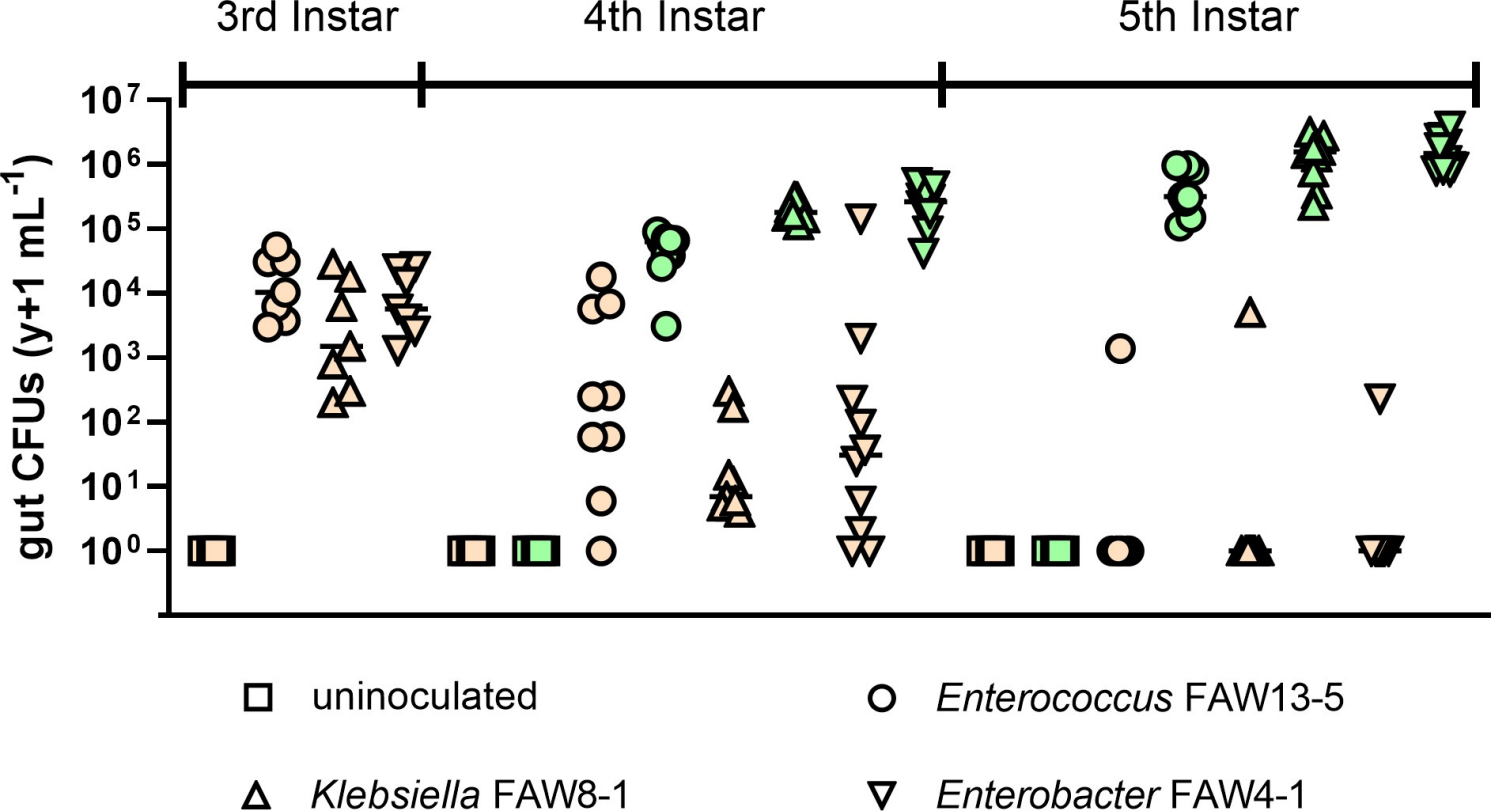
Impact of bacterial isolates on fall armyworm gut colony-forming units (CFUs). Each symbol represents a single individual at each treatment and developmental time (n = 7–9). Insects were inoculated with bacteria at the beginning of the second instar on wheat germ artificial diet until the third instar, before being transferred to new wheat germ diet (beige) or gamma-irradiated corn leaves (green). Insects that hatched from surface-sterilized eggs and had not been fed bacteria (uninoculated) did not have detectable gut bacterial CFUs. Insects fed bacteria and maintained on artificial diet had smaller and more variable bacterial populations compared to those feeding on corn. The bacterial titers increased through the development of fall armyworm larvae to the last instar.

The *Enterococcus*, *Klebsiella*, and *Enterobacter* isolates we initially acquired from field-collected larvae [15] established at approximately equal titers in third instar fall armyworm larvae (Fig 2; H = 3.602; p = 0.165). Axenic larvae that were treated with sterile PBS did not yield any detectable bacteria in the gut of the larval instars when fed on either the wheat germ or gamma-irradiated corn diets. Microbial titers in insect guts followed two separate trajectories: bacterial populations increased in corn-fed insects, while insects maintained on wheat germ diet had sharp decreases in abundance of gut bacteria over the course of the experiment (p < 0.001). Insects fed on corn had higher titers of gut bacteria and less variability between individuals compared to those fed on wheat-germ diet. Insect developmental stage had a significant impact on the microbial titers, where larger insects had greater gut bacterial populations. While we did not observe differences between the titers of *Klebsiella sp*. FAW8-1 and *Enterobacter sp*. FAW4-1 in 4^th^ or 5^th^ instars feeding on corn, *Enterococcus sp*. FAW13-5 had lower titers than both in 4^th^ instar larvae (p < 0.007) and *Enterobacter* in 5^th^ instar larvae (Z = 2.73; p = 0.020).

### Influence of pinto bean artificial diet on *Enterobacter* proliferation

**Fig 3:**
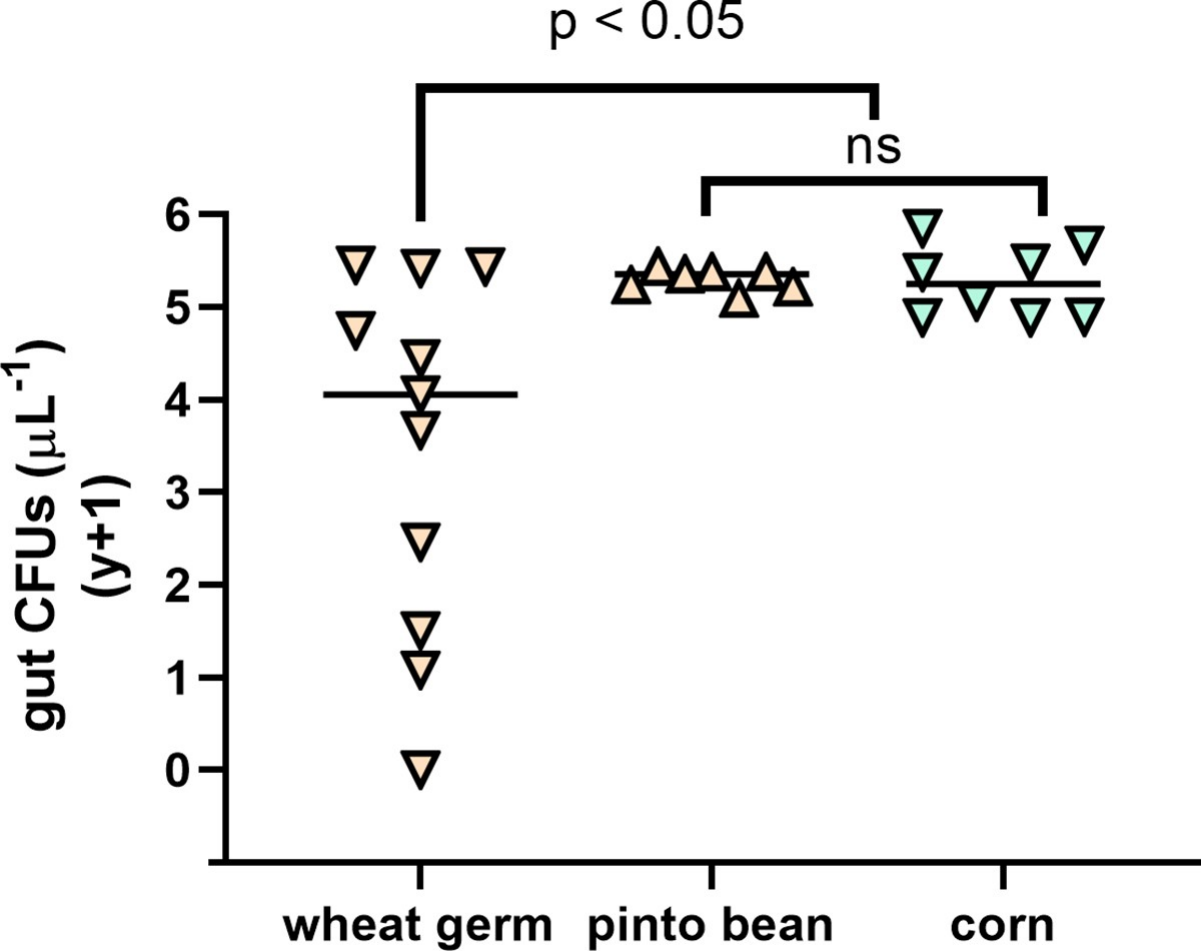
Impact of wheat germ artificial diet, pinto bean artificial diet, and corn leaf diet on *Enterobacter sp*. FAW4-1 colony-forming units (CFUs) in the gut of fall armyworm (n = 8–11). Insects were maintained on their respective diet after inoculation through the second instar. Each symbol represents a single individual. Data were analyzed using an ANOVA with log_10_(*y*+1) transformed values. No gut bacteria were detected in the guts of axenic larvae that were not treated with bacteria and grown on respective diets (not shown).

We used a second diet formulation to compare to our results from sterile wheat germ diet. As observed in our first experiment, there was a significant difference in *Enterobacter* titers between insects feeding on gamma-irradiated corn versus wheat germ diet (Fig 3; p = 0.034). We also observed greater variation in bacterial titers in larvae fed on wheat germ diet compared to both pinto bean diet and corn foliage. Notably, the decrease in microbial titers was not as rapid as was observed in our first experiment. However, the pinto bean artificial diet produced results similar to the corn-fed insects in both the population titers as well as the variance between individuals. As with the corn diet, the pinto bean diet resulted in microbial populations that were significantly higher than larvae fed on wheat germ diet (p = 0.037).

### Impact of insect host species on diet–bacteria interactions

**Fig 4:**
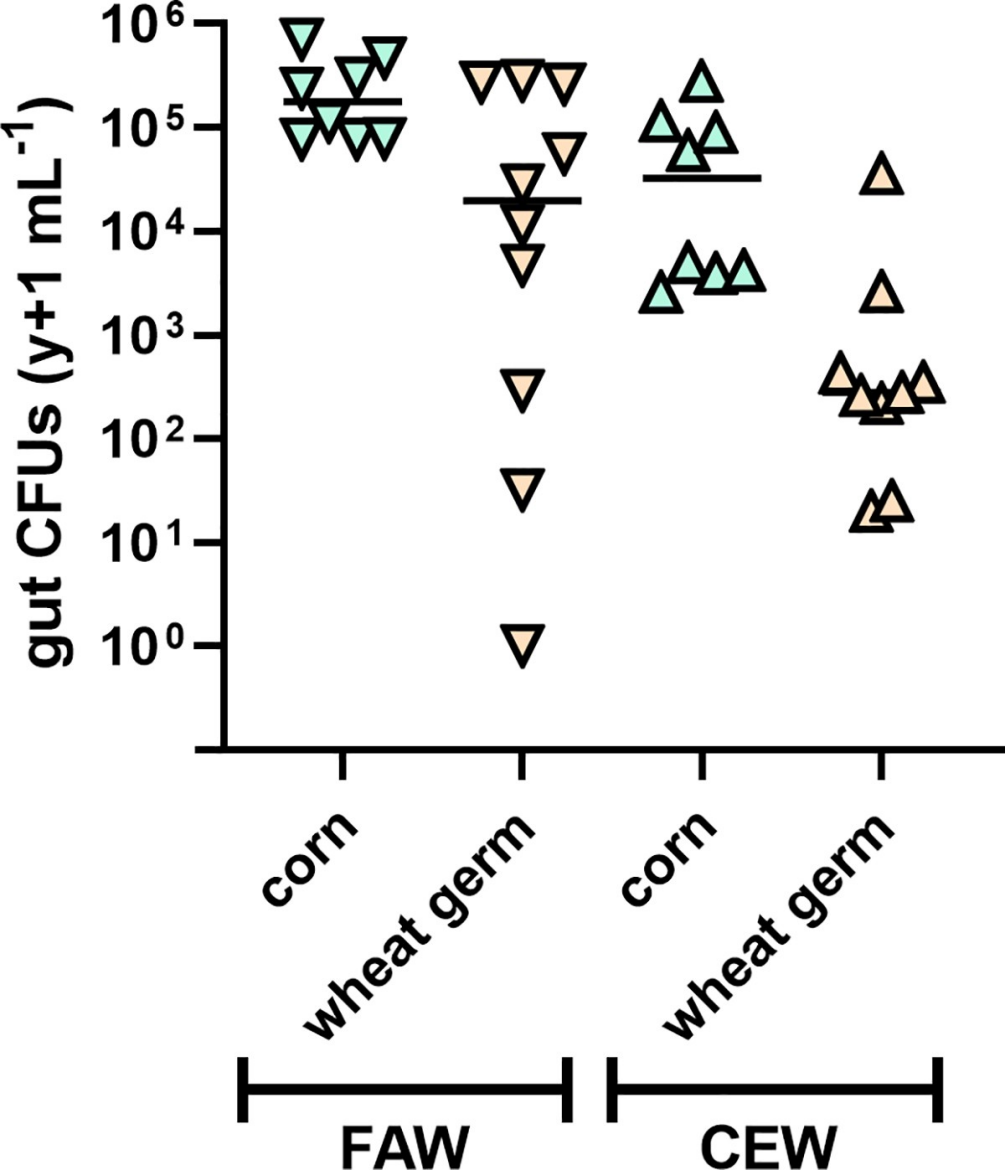
Impact of corn and wheat germ artificial diet on proliferation of *Enterobacter sp*. FAW4-1 (colony forming units (CFUs)) in larval guts of corn earworm (CEW) and fall armyworm (FAW). Each symbol represents a single individual (n = 8–11). Insects were maintained on their respective diet following one bacterial inoculation. Axenic larvae that were not treated with bacteria and grown on respective diets (not shown) did not yield detectable microbes for either species on any diet at the completion of the experiment (fourth instar).

We conducted a third experiment with wheat germ artificial diet and corn leaves to compare *Enterobacter* titers between fall armyworm and corn earworm. Gut bacterial populations followed similar trajectories in both corn earworm and fall armyworm (Fig 4). Each insect species had higher *Enterobacter* gut populations when fed on corn compared to those fed on wheat germ diet (p < 0.025). Similar to what we observed in prior experiments, there was less variation in the gut bacterial populations for both species fed on corn compared to wheat germ diet.

**Fig 5:**
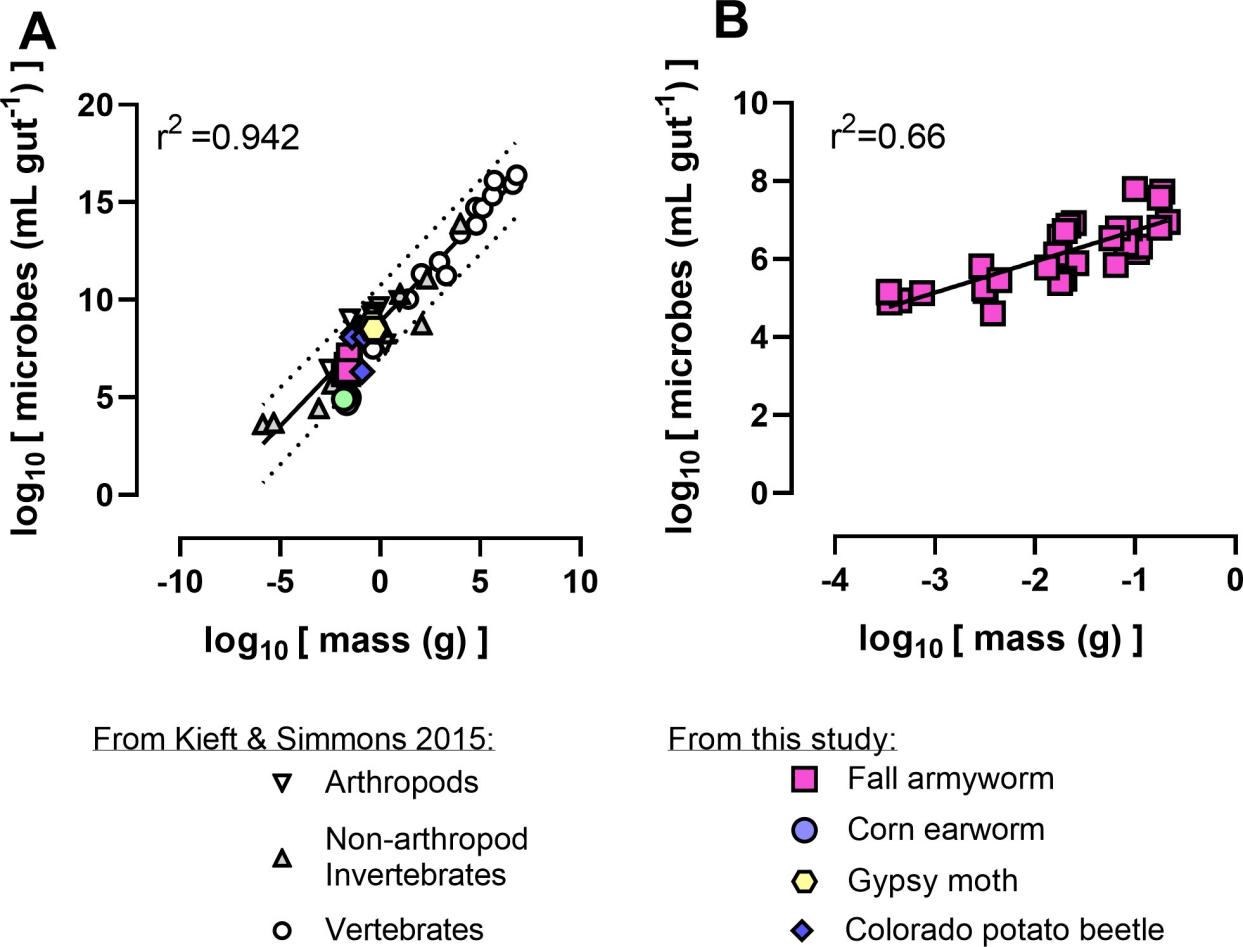
Relationships between animal biomass and the number of associated microbes in fall armyworm, corn earworm, gypsy moth, Colorado potato beetle, and other animals (A). Microbial biomass in fourth instar larvae were used for these leaf-feeding insects. We observed significant positive relationships when we investigated microbial titers across larval developmental stages of fall armyworm consuming corn (B). Solid and dotted lines correspond to a linear regression and 95% prediction intervals in panel A were from data originally published in Kieft and Simmons ([34]. Relationships in microbial titers versus fall armyworm biomass (B) indicate that body size changes alter microbial titers through development in larvae fed corn. The regression slopes differ slightly between the two panels; the regression including all major animals had a slope of 1.07 (95% confidence interval 0.97–1.17), while the regression including only fall armyworm had a shallower slope of 0.80 (95% confidence interval 0.58–1.01).

We compared the body mass of individual 4^th^ instar corn earworm, fall armyworm, gypsy moth, and Colorado potato beetle larvae fed on plants with that of other animals [34]. We found that for the species we tested, the relationship between microbial titers and body masses fell within the 95% prediction interval of previously published literature (Fig 5A). Moreover, we observed a significant, positive correlation between fall armyworm body size and microbial titer across larval development (Fig 4B; F_1,30_ = 60.0 p < 0.001). However, when we analyzed the body mass–microbial titer relationships within a given larval instar, we did not observe significant relationships for corn earworm, fall armyworm, gypsy moth, or Colorado potato beetle, indicating that a greater spread of body sizes is needed to address these dynamics.

## Discussion

Multiple intersecting factors can influence insect gut microbiomes, including host environment, colonization by diverse members, and ingestion of different diets having different phytochemical and nutrient compositions. In this study, we demonstrated that a single bacterial introduction is sufficient for bacterial establishment and sustained proliferation in lepidopteran guts. Bacteria populations were able to persist through larval molts, suggesting that these bacteria can establish in the gut and are not merely transient associates. However, bacterial population dynamics were strongly influenced by the diet the insect consumed. In both fall armyworm and corn earworm, we found that gut bacterial populations responded differently to diets of corn foliage or wheat germ artificial diets. Bacterial isolates established and proliferated through the development of caterpillars fed on corn leaves. In contrast, insect–microbial relationships were destabilized when the larvae fed on wheat germ diet, in some cases eliminating the isolate from the gut. When we provided insects with a semi-artificial diet (pinto bean diet) formulation with less accessible nutrients, bacterial titers were similar to those in plant-fed larvae. Our results show that microbial colonization and proliferation are dynamic processes in the lepidopteran gut that can be in part driven by diet provided to larvae.

The lepidopteran larval gut system is noteworthy for both its simplicity and its physicochemical properties. The midgut is a morphologically simple tube that does not possess any clear morphological modifications, enlargements, or connections, which can be found in some other insect orders [22]. Additionally, the midgut lumen pH in lepidopterans is usually alkaline; both of the species we used here have reported midgut lumens exceeding pH 9.5 [24,25]. These host traits, coupled with some studies showing overlap between microbes found in guts and foliar diets, have fueled speculation that the microbes associated with caterpillars are entirely ephemeral and transient [17]. Our results do not support the assertion that the associated microbiota is simply a component of the macerated foliage passing through the insect’s digestive system. Rather, the bacterial populations that establish in lepidopteran guts can persist through larval development. Our results indicate that bacteria not only have physiological tolerances to the alkaline gut environment but may also extract suitable nutrition from the macerated food bolus to sustain populations.

In contrast to the corn and pinto bean diets, caterpillars feeding on wheat germ artificial diet had decreased bacterial titers, which may be a result of one or more components of the wheat germ diet. First, wheat germ possesses the lectin agglutin, and lectins can have antibiosis activities against a variety of microorganisms and insects [35–37]. Second, the wheat germ diet we employed contains a high amount of soluble carbohydrates and protein while, in contrast, pinto bean diet was more restrictive and resembled plant tissues. Notably, wheat germ also contains multiple components that are derived from microbial sources (yeast nutrient). It is unclear if and how carbohydrate and/or protein complexity of diets may encourage host–microbe associations in these systems but warrants further exploration. Finally, there could be micronutrients that promote bacterial growth in corn and pinto beans that are not present in the wheat germ diet. While wheat germ diet is optimized for insect rearing, it is potentially a poor substrate for evaluating insect–microbe interactions for Lepidoptera, depending upon the species and diet formulation. Other studies have noted a reduced number of microbes in guts of closely related species feeding on wheat germ diets [38], so what we observe here could be common. Yet there are some lepidopterans that seem to have well-established microbial gut populations when provided wheat germ-based diets [6,10,12]. Even within our study, we observed variation in bacterial titers on wheat germ diets between experiments (see Figs 1 and 2). Our results suggest that caution should be taken in connecting insect–microbial interactions on artificial diet to interactions occurring in nature, because diet may skew both bacterial gut populations and communities.

Since pinto bean diet induced similar colonization patterns as corn leaves, it may serve as a suitable and manipulatable substitute for wheat germ diet and as a tool for exploration of microbial function in these systems. Not only does pinto bean diet provide a media that allows for manipulation under sterile conditions, but it also provides a more consistent dietary source to the larvae through time. Artificial diets that include more recalcitrant plant-based components that exclude the nutrient rich components common in Lepidoptera diet formulations may serve as similar experimental tools as the pinto bean diet we described here.

We observed a positive relationship between growth allometry and the microbial biomass present in the folivores we assayed. When we evaluated bacterial titers for plant-fed corn earworm, fall armyworm, gypsy moth, and Colorado potato beetle, we observed relationships within predicted values derived for other animal species [34]. Moreover, we observed a positive relationship between microbial titer and animal biomass for different fall armyworm instars feeding on plants. Prior work in lepidopterans has found a positive relationship between growth and microbial titer [8], while others have not [17]. These discrepancies may be due to sampling methods or system-specific attributes. It should be noted that our experiments were limited to the larval life stages, and the species we used undergo radical anatomical changes during metamorphosis, especially of their digestive systems. For lepidopterans, the gut system becomes dramatically reduced compared to the rest of the body, with a greater portion of their body mass being invested in reproductive systems. The strength of the relationship between growth allometry and microbial titer may change through these developmental stages where there could be inflection points between life stages that become constant or negative trending.

While we observed strong interactions between gut microbes and plant diet in our study, we still have no clear indication of physiological cooperation between the gut microbes and the herbivore host. Currently, there is relatively weak evidence for nutritional benefits provided by bacteria in lepidopterans [18,19]. However, the persistence of the isolates we used in our system suggests that some metabolic exchanges between the host and associated microbes may be present. For example, stimulation of immune-related responses to the presence of bacteria in lepidopterans has been reported, particularly when complex diets or plants are involved [30,39]. In the same vein, bacteria in these systems may have antimicrobial properties that limit the establishment of some pathogens [40]. The fact that we observed increases in the microbial populations of larvae feeding on plants suggests there may be systemic metabolic changes in the host; evaluating metabolic shifts by bacteria would provide a better basis for how these organisms are interacting and determine if they contribute to nutrient provisioning or hormone signaling.

Overall, there continue to be knowledge gaps in the microbial and/or host traits that enable colonization of lepidopteran hosts, and our results indicate that these are dynamic processes that should explored. These results, coupled with recent work indicating that diet does not explain variation observed in the gut microbiome of some caterpillar species [41], suggest that all microbes associated with lepidopterans are not simply transient associates. However, there is an overarching complexity to host–microbe interactions in this order that requires further elucidation, including studies into the basic physiological functions of these relationships.

## Supporting information

S1 Fig Uncropped gel image used in Fig 1. (JPG)

S1 Table Underlying data used for the analyses in this paper. (XLSX)
