# Supplementary figures and images for: Diet influences proliferation and stability of gut bacterial populations in herbivorous lepidopteran larvae
Source: PLoS One. 2020 Mar 13;15(3):e0229848. doi: 10.1371/journal.pone.0229848 (PMC7069608; doi:10.1371/journal.pone.0229848)

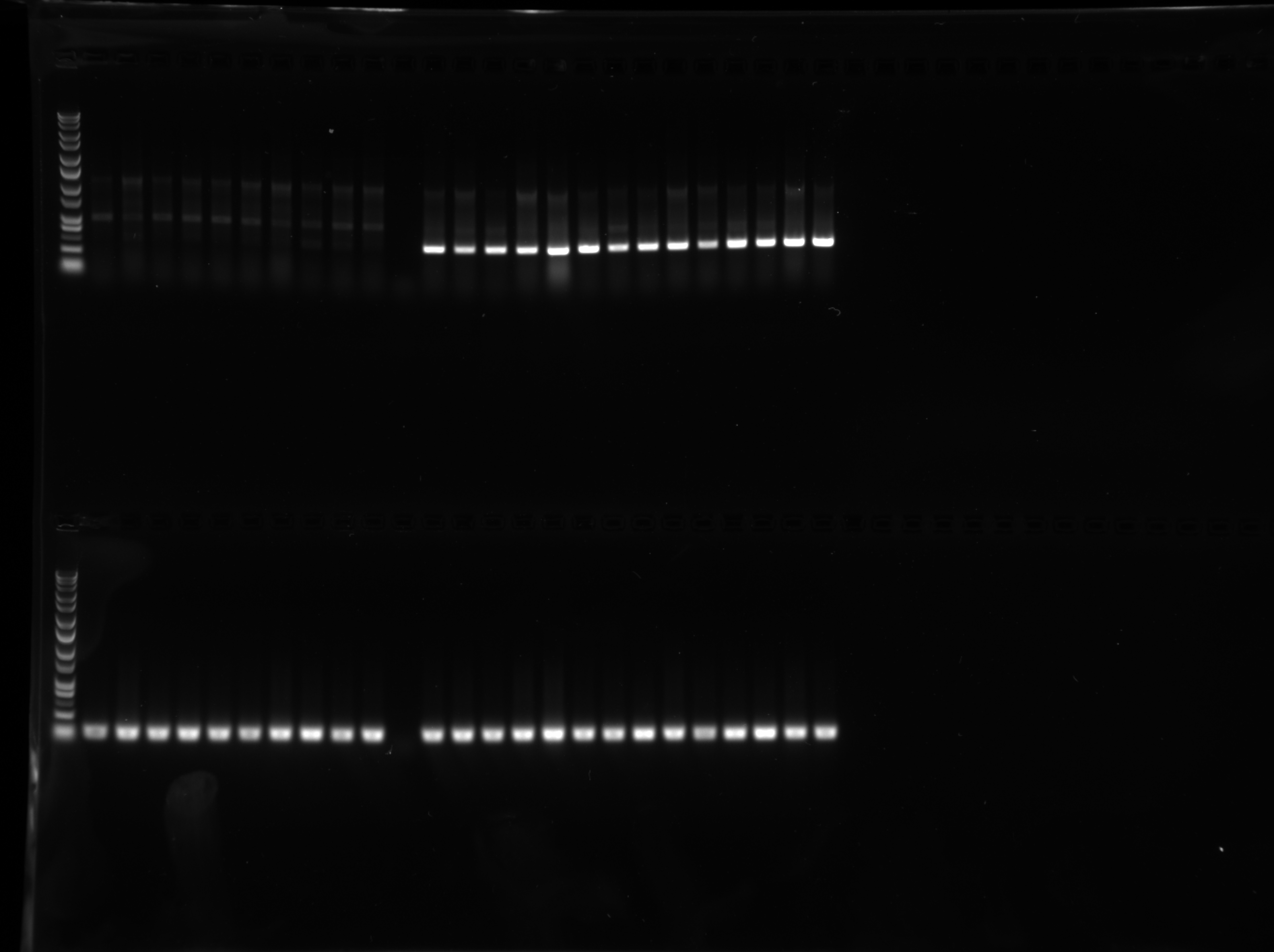

Supplement: S1 Fig — (JPG) [file pone.0229848.s001.jpg]
